# *Wolbachia* Horizontal Transmission Events in Ants: What Do We Know and What Can We Learn?

**DOI:** 10.3389/fmicb.2019.00296

**Published:** 2019-03-06

**Authors:** Sarah J. A. Tolley, Peter Nonacs, Panagiotis Sapountzis

**Affiliations:** ^1^Department of Ecology and Evolutionary Biology, University of California, Los Angeles, Los Angeles, CA, United States; ^2^Centre for Social Evolution, University of Copenhagen, Copenhagen, Denmark

**Keywords:** symbiosis, fungus-growing ants, horizontal transmission, social interactions, *Wolbachia*

## Abstract

While strict vertical transmission insures the durability of intracellular symbioses, phylogenetic incongruences between hosts and endosymbionts suggest horizontal transmission must also occur. These horizontal acquisitions can have important implications for the biology of the host. *Wolbachia* is one of the most ecologically successful prokaryotes in arthropods, infecting an estimated 50–70% of all insect species. Much of this success is likely due to the fact that, in arthropods, *Wolbachia* is notorious for manipulating host reproduction to favor transmission through the female germline. However, its natural potential for horizontal transmission remains poorly understood. Here we evaluate the fundamental prerequisites for successful horizontal transfer, including necessary environmental conditions, genetic potential of bacterial strains, and means of mediating transfers. Furthermore, we revisit the relatedness of *Wolbachia* strains infecting the Panamanian leaf-cutting ant, *Acromyrmex echinatior*, and its inquiline social parasite, *Acromyrmex insinuator*, and compare our results to a study published more than 15 years ago by Van Borm et al. (2003). The results of this pilot study prompt us to reevaluate previous notions that obligate social parasitism reliably facilitates horizontal transfer and suggest that not all *Wolbachia* strains associated with ants have the same genetic potential for horizontal transmission.

## Introduction

*Wolbachia pipientis* is a maternally inherited α-proteobacterium widely found in arthropods (Werren et al., 2008). *Wolbachia* exhibits reproductive parasitism in most arthropod species by manipulating the reproductive physiology of hosts and inducing female-biased sex ratios via one of four mechanisms: cytoplasmic incompatibility, feminization of genetic males, male-killing, or enforcing parthenogenesis (for reviews see Stouthamer et al., 1999; Werren et al., 2008). Although transmission within species is strictly vertical, *Wolbachia* phylogenies rarely correspond to host phylogenies, suggesting horizontal transmission (HT) also occurs (Zhou et al., 1998; Vavre et al., 1999; Raychoudhury et al., 2009; Stahlhut et al., 2010; Ahmed et al., 2013).

Horizontal transmission of intracellular bacterial symbionts require intimate tissue-level interaction between current and future hosts; predator–prey and host–parasitoid relationships have therefore been proposed to explain observed HT events (e.g., Heath et al., 1999; Noda et al., 2001; Yang et al., 2013; Carvalho et al., 2014; Ahmed et al., 2015; Mascarenhas et al., 2016). Ants are the only lineage of social Hymenoptera where permanent social parasites, closely related to their host, commonly invade mature colonies (Boomsma et al., 2014). As ants are perennial, intimate inquiline cohabitation where social parasites live with hosts across generations offers ample opportunities for HT. This idea was first explored in *Acromyrmex echinatior* colonies, which are considered closed systems for endosymbionts since workers are highly aggressive toward non-nestmates (Larsen et al., 2014). Colonies can, however, be infiltrated by socially parasitic *Acromyrmex insinuator* queens, which invade and adopt the host colony odor (Lambardi et al., 2007; Nehring et al., 2015). Van Borm et al. (2003) first suggested that HT events occur between *Wolbachia* endosymbionts of *A. echinatior* and *A. insinuator* based on shared bacterial genotypes between cohabiting ant species. Later research suggested the same for *Solenopsis daguerrei*, a social parasite of *S. saevissima* (Dedeine et al., 2005; Martins et al., 2012) and for another fungus-growing ant, *Sericomyrmex amabilis*, and its social parasite *Megalomyrmex symmetochus* (Adams et al., 2013; Liberti et al., 2015).

In this perspective, we summarize research that has investigated *Wolbachia* HT events in ants, examine limitations of methods and study systems used, and propose future research. We also partially repeat one of the first field studies (Van Borm et al., 2003) characterizing *Wolbachia* endosymbionts of two fungus-growing ant species: the leaf-cutting ant, *A. echinatior*, and its social parasite, *A. insinuator*. Our pilot results, originating from a single *A. echinatior* colony parasitized by three *A. insinuator* queens, only partially confirmed these earlier findings. This highlights the importance of re-evaluating past and current methods and redirecting future efforts to include whole genome sequencing (WGS) data, which could increase the resolution of phylogenetic relationships and reveal pioneering insights into the genes and mechanisms that allow *Wolbachia* to jump to new hosts.

## Methods

A single *A. echinatior* queen and three parasitic *A. insinuator* queens cohabiting a mature colony (Ae724; collected in Gamboa, Panama, May 2015) were isolated in separate sterile petri dishes (similar to Stürup et al., 2014). After a 36-h period, ca. 40 eggs were collected from each queen and stored at −20°C. DNA was extracted using the DNeasy Tissue Kit (Qiagen) and a 603 bp region of the *Wolbachia* surface protein (*wsp*) was amplified using 81F/691R primers (Braig et al., 1998) and PCR conditions as described in Baldo et al. (2006b). PCR products were purified using the Invitek PCR purification kit, cloned using the TOPO TA cloning kit (Invitrogen, United States), and 24 colonies from each cloning were sent for Sanger sequencing (MWG, Germany). We checked chromatographs and removed primer sequences using Geneious (v. 9.0.4). Trimmed sequences (MG547478-MG547559) were queried against the non-redundant NCBI database to compile the top 100 hits. All sequences were aligned with ClustalW, sites with gaps were removed and sequences that could not align to the entire 426 bp reduced alignment were removed. Maximum Likelihood phylogenetic trees with 1,000 bootstrap iterations and the TVM+G model (jmodeltest v2.1.7) were run in Garli version 2.01.1067 (Zwickl, 2006). A consensus tree was configured in Geneious v9 (Kearse et al., 2012), and one representative *wsp* sequence from the same host species (>99%) was picked. The tree was further modified in FigTree v1.4.3 (Rambaut, 2016). As described in Baldo et al. (2006b), the strain profiles for each *wsp* sequence from this study was identified based on four conserved hypervariable regions (HVR) (Supplementary Tables S1, S2). Since eggs were pooled for sampling, coinfecting strains present in each species may not occupy the same individuals.

## Results

**FIGURE 1:**
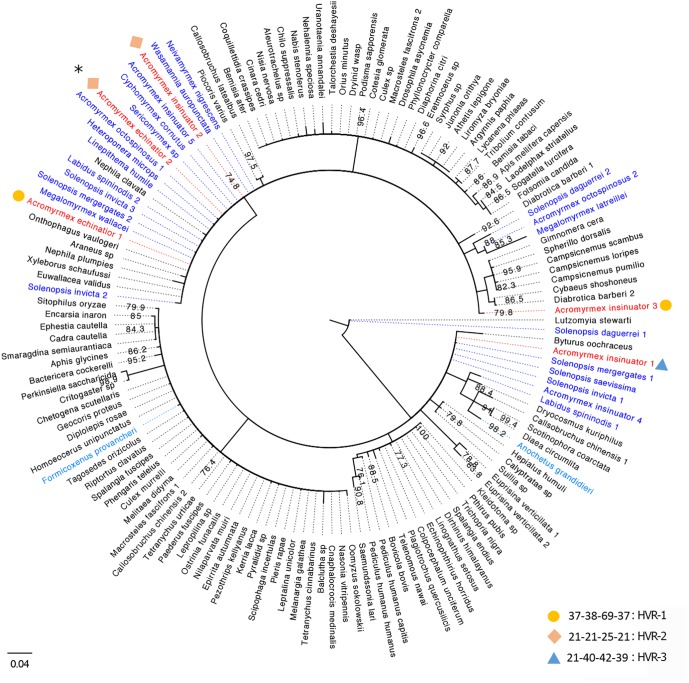
Consensus tree for *Wolbachia* strains based on the *wsp* gene. Strains are represented by the infected arthropod host species with which they are associated. Host names based on sequences generated in the current study are colored red and those from previous studies on ant species are colored blue (Neotropical species in dark blue, two others in light blue). Sequences from *Wolbachia* strains associated with non-ant hosts are presented in black font and bootstrap support is shown at each node. In total, 24 unique *Wolbachia* sequences from ant hosts and 112 sequences from non-ant arthropod hosts were downloaded from GenBank and used in this phylogenetic analysis. Numbers next to species names represent genetically distinct strains harbored in the same species. Information about the HVR barcoding is given next to each of the *Wolbachia* strains identified in our study using circles, diamonds, and triangles (legend bottom right), for details see Supplementary Tables S1, S2. Since HVR barcoding was only analyzed for the 83 *wsp* sequences from the four *Acromyrmex* queens used in this study, only the representative strains from our study in red font are given a corresponding HVR type. Strains matching the Van Borm et al. (2003) isolates, B2 and B1, are labeled here as *Acromyrmex insinuator* 4 and 5. The majority of BLAST hits clustering with the *A. echinatior* and *A. insinuator* sequences generated in this study are *Wolbachia* strains from Neotropical New World ant species. The only BLAST hits from ant hosts that are not Neotropical New World ant species were phylogenetically isolated (light blue species; *Formicoxenus provancheri*, occurring in North America, and *Anochetus grandidieri*, a species endemic to Madagascar). The asterisk at the top left of the figure marks the HVR-2 strain that is widespread among mainly ants, but also other insect hosts in the Americas.

Van Borm et al. (2003) originally characterized nine *Wolbachia* infections: two strains in *A. echinatior*, four in *Acromyrmex octospinosus*, and three in their social parasite, *A. insinuator*. Some strains were specific to *Acromyrmex* ant species (A1 and B2), while others were present across multiple species (B1 and Bcons). Considering this earlier study was conducted >15 years ago, we reconstructed the phylogenetic relationships of previously identified *wsp* sequences (Van Borm et al., 2003), *wsp* sequences generated in our pilot study (from one host and three cohabiting parasitic queens), and closely related *wsp* sequences available on NCBI from other arthropod hosts (Figure 1). Using similar methods as Van Borm et al. (2003) (with the exception of extracting DNA from eggs rather than gynes), we identified three *wsp* genotypes named HVR1-3 (Supplementary Tables S1, S2). While HVR-1 was the dominant *A. echinatior* strain in our study (Supplementary Figure S1), it was not identified in the previous study. HVR-2 was identical (>99%) to strain B1 (AF472563; Van Borm study). We identified HVR-2 in both *A. insinuator* and *A. echinatior* while Van Borm et al. (2003) found HVR-2 only in *A. insinuator* and a closely related but not identical strain (Bcons) in *A. echinatior*. HVR-2 has also been found in *A. octospinosus* (Van Borm et al., 2003; Andersen et al., 2012). HVR-3 was identical (>99%) to strain B2 (AF472560; Van Borm study) and, as before, was only found in *A. insinuator*. Two strains identified before (AF472558-9) were not found in the colony we analyzed. The Van Borm study suggested multiple HT events occurred for *Acromyrmex* ants to acquire their *Wolbachia*, as evidenced by their findings showing distantly related *Wolbachia* strains shared by closely related *Acromyrmex* hosts and the reverse, closely related *Wolbachia* present in distantly related host species. Our results were consistent with the Van Borm study where *wsp* sequences from *Acromyrmex* hosts were most similar to those from distantly related *Solenopsis* fire ant hosts. Our new phylogeny also revealed additional ant hosts harboring closely related *wsp* sequences. HVR-2 seemed the most cosmopolitan strain in ant hosts as it is present in at least nine ant genera (Figure 1).

## Discussion

**FIGURE 2:**
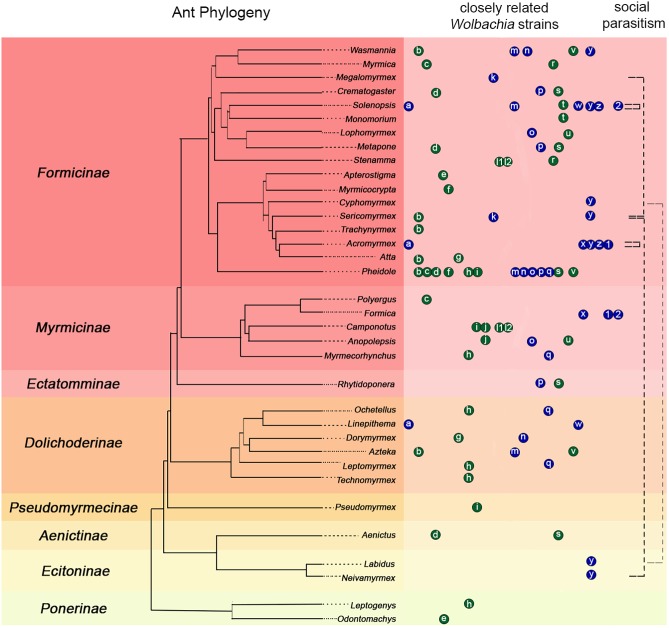
Compilation of previous literature including ant phylogenies, *Wolbachia* infections, strain typing, and known connections between social parasitism and shared *Wolbachia* strains. The ant phylogeny on the left was constructed using data from Moreau et al. (2006), Schultz and Brady (2008), and Branstetter et al. (2017). Ant subfamilies are presented on the left of the phylogeny with colored backgrounds separating them. Colored circles with small case letters on the right of the ant phylogeny connect ant hosts suspected to carry similar *Wolbachia* strains based on previous phylogenies using the *wsp* gene (blue) or MLST typing (green). Letters in the circles indicate the published source where authors built phylogenies that showed potentially common *Wolbachia* strains in different host ant species: a: (Dedeine et al., 2005); b–j: (Frost et al., 2010); k: (Liberti et al., 2015); l1: (Ramalho et al., 2017); m–q: (Rey et al., 2013); h, l2, r–v: (Russell et al., 2009); w: (Fernando de Souza et al., 2009); x: (Tsutsui et al., 2003; Reuter et al., 2005); y: (this study); z: (Van Borm et al., 2003); 1, 2: (Viljakainen et al., 2008). Each letter circle occupying the same column represents a set of highly similar *Wolbachia* strains defined as belonging to the same clade (or a potentially common identical strain) according to the corresponding source publication. Circle order is alphabetical and distances between circles are not indicative of the genetic similarity of strains. Therefore, some heavily sampled genera, such as *Pheidole*, may have the same strain represented in more than one publication in which different ant genera were analyzed. The black dashed lines in the rightmost column connect known instances of social parasitism between ants based on previous literature (see text for details). Black dashed lines that start and end in the same genus highlight that *Solenopsis* and *Acromyrmex* have social parasites within their genera and they share identical *Wolbachia* strains with them (Van Borm et al., 2003; Dedeine et al., 2005; Martins et al., 2012; our study). The light gray dashed line connecting *Labidus* and *Cyphomyrmex* highlight an almost identical shared *Wolbachia* strain (differing only by 1 bp; Figure 1). However, there is no data suggesting *Labidus* predates *Cyphomyrmex* colonies.

HT events are believed to have largely contributed to the *Wolbachia* pandemic, where an estimated 50–70% of all insect species are infected (Werren et al., 2008; Saridaki and Bourtzis, 2010; Weinert et al., 2015). High frequency of phylogenetic incongruences between hosts and *Wolbachia* strains (as seen in Figure 2) suggests HT events are relatively common on an evolutionary time scale despite the fact that they are difficult to predict and observe in nature. The results of our pilot experiment support the hypothesis that HT has occurred between *A. echinatior* and its social parasite, *A. insinuator*, originally proposed by Van Borm et al. (2003). As in the Van Borm study, we found distantly related *Wolbachia* strains occupying the same host (HVR-2 and -3 in *A. insinuator*) as well as identical strains occupying distantly related hosts (HVR-2; Figure 1, 2). Although social parasitism should provide ample opportunity for HT, our results suggest some strains, like HVR-2, may be better equipped to “jump” between hosts. Although much about HT remains unknown, minimum conditions must be fulfilled for HT to occur: (1) there must be suitable environmental conditions (in the new host as well as the medium/environment the bacteria transitions through), (2) the bacterial strain must have the genetic potential for transfer, and (3) there must be a mechanism that will mediate the HT event.

### Part 1: *Wolbachia* Genetic Potential

*Wolbachia* can be artificially transferred across insect genera in the lab (e.g., Zabalou et al., 2004; Hoffmann et al., 2011) and following transfers, adaptations to new hosts may rapidly occur (McMeniman et al., 2008). This ability to invade new hosts is consistent with the identification of genetically similar strains in taxonomically unrelated hosts (e.g., Heath et al., 1999; Raychoudhury et al., 2009). *Wolbachia* is obligately intracellular yet is capable of surviving extracellularly for several months before reinvading new cells and establishing a stable infection (Rasgon et al., 2006). Although mechanisms of natural HT remain elusive, *Wolbachia* has demonstrated the ability to successfully “jump” across cells, cross somatic tissues, and reach reproductive organs (Frydman et al., 2006; White et al., 2017). Successful transfers may be attributed to the bacterium’s ability to adapt to new environments. This could be accomplished by recombination, likely mediated by inactive bacteriophages introducing “exotic genes,” resulting in gene gains and diversification of the bacterium’s genome (Wu et al., 2004; Klasson et al., 2009; Vos and Didelot, 2009; Ellegaard et al., 2013). Indeed, the *Wolbachia* genome has a high number of repetitive elements and ankyrins, mostly introduced by bacteriophages (Ishmael et al., 2009; Kent and Bordenstein, 2010; Leclercq et al., 2011; Siozios et al., 2013). While the function of these gene gains has not been fully deciphered, genomic comparisons with a mutualistic strain infecting nematode hosts, *w*Bm (Foster et al., 2005), suggest they play a role in the bacterium’s ability to induce reproductive phenotypes in arthropods.

Considering the significant genomic differences and tissue tropisms between *Wolbachia* strains, we expect not all strains have the same potential for transmission. For example, while *Wolbachia* is typically localized in the reproductive tract (e.g., *w*Mel, *w*Sty), there are some B-group strains that colonize somatic (non-reproductive) tissues (e.g., *w*No, *w*Ma; Veneti et al., 2004). As expected, not all strains can survive a transfer or induce reproductive phenotypes necessary to facilitate its spread in new host populations (Zabalou et al., 2008; Veneti et al., 2012). Phylogenetic comparisons using *wsp* sequences (Van Borm et al., 2003; Figure 1) also suggest that one of the strains in *Acromyrmex* (HVR-2) may have a greater propensity for HT than HVR-1 and HVR-3. HVR-2 is not only common across the Panamanian *Acromyrmex* species (*A. echinatior*, *A. insinuator*, *A. octospinosus*), where it has been identified as *w*SinvictaB (Andersen et al., 2012), but also in ant hosts across four subfamilies (Figure 1). In contrast, HVR-1 and HVR-3 appear specific to their respective host species and are far more dominant in those hosts than the shared HVR-2 (Supplementary Figure S1). This distribution suggests that HVR-1 and HVR-3 are better adapted to their respective host species while HVR-2 is a generalist capable of infecting hosts with diverse life histories. Interestingly, HVR-2 (*w*SinvictaB) appears to be dominant in *A. octospinosus* (Andersen et al., 2012), but occurs as either a single or double infection with the rare and sparse *w*SinvictaA (Andersen et al., 2012).

### Part 2: Potential Transmission Routes in Ants

Ant sociality offers ample opportunities for *Wolbachia* transfer across hosts and may be especially favorable for species prone to interspecific social interactions or with less restrictive tissue tropisms. For example, fungus-growing ants are a host where *Wolbachia* has uncommon tissue tropism; it is present extracellularly in the gut lumen and may reach high titers in the hemolymph (Andersen et al., 2012; Frost et al., 2014; Sapountzis et al., 2015). A common resource, such as a fungal garden, may thus facilitate HT of *Wolbachia* strains between cohabiting *A. echinatior* and *A. insinuator*, as the ants deposit their feces in the fungus, feed on it, and cover their brood with it (which also feeds on the fungus). Similarly, an identical *Wolbachia* strain has been found between a workerless social parasite, *S. daguerrei*, and its host ant species *S. invicta* (Dedeine et al., 2005). However, a shared *Wolbachia* strain was not found between *M. symmetochus* social mercenaries and its host, *S. amabilis*, suggesting cohabitation does not always result in HT (Liberti et al., 2015).

Inquiline mites may also have the capacity to vector *Wolbachia* between attine species cohabiting the same nest or foraging on the same plants. However, mites in *Acromyrmex* nests appear to be saprophytic, not parasitic (Peralta and Martínez, 2013), making this alternative transmission route unlikely. Parasitic phorid flies could also serve as a common vector between all three ant species (Brown and Feener, 1998; Fernández-Marín et al., 2006; Pérez-Ortega et al., 2010; Guillade and Folgarait, 2015), however, so far there is no data suggesting they have contributed to HT events (Dedeine et al., 2005).

Independent of being intra- or extra-cellular symbionts, HT may also be mediated by predators such as *Neivamyrmex*, a genus of army ant known to raid nests of fungus-growing ants and consume their brood (Lapolla et al., 2002; Powell and Clark, 2004). Army ant taxa (subfamilies Aenictinae, Dorylinae, and Ecitoninae) are often infected with *Wolbachia* and thus offer exciting opportunities for studying potential HT (Figure 2). HVR-2 is distributed across species from the subfamilies Myrmicinae (*Acromyrmex* and *Sericomyrmex*) and Ecitoninae (*Neivamyrmex*; Figure 1, 2). Similarly, an identical *Wolbachia* strain is shared between *Cyphomyrmex* and army ants of the genus *Labidus* (subfamily Ecitoninae; Figure 1), however, there is no known data confirming whether these army ants attack fungus-growing ants (Figure 2).

### Part 3: Genomic Data and Sampling Power Limitations

*Wolbachia* strain typing has relied on several different genes, one of them being the 16S rDNA gene used when performing targeted sequencing (e.g., Kautz et al., 2013; Ramalho et al., 2017). This method is not appropriate to build phylogenies as the 16s gene is highly conserved and cannot distinguish closely related *Wolbachia* strains (Andersen et al., 2012). The *wsp* gene has also been used extensively for *Wolbachia* characterization because its rapid sequence evolution enables differentiation between closely related strains and it contains four HVRs useful in solidifying strain identification (Baldo et al., 2006b). However, the relatively short sequence length (<600 bp), high recombination rate (Baldo et al., 2005) and, in some arthropod hosts, strong positive selection (Jiggins et al., 2002), make *wsp* suboptimal for constructing phylogenies. Nevertheless, the *wsp* gene remains a useful “quick and dirty” approach to distinguish phylogenetic relationships of *Wolbachia* strains and is, in most cases, the only sequence available to build phylogenies. Due to these limitations, multilocus sequence typing (MLST) was introduced, which uses concatenated alignments of five housekeeping genes (Baldo et al., 2006a; Bordenstein et al., 2009). However, due to frequent recombination, WGS is the only accurate method to infer phylogenetic relationships (Bleidorn and Gerth, 2018).

A particular challenge to studying the evolutionary relationships of *Wolbachia* in arthropods is that hosts are frequently infected with multiple strains (Hiroki et al., 2004; Mouton et al., 2004; Frost et al., 2010; Andersen et al., 2012; Zhao et al., 2013), making even MLST and WGS approaches exceedingly challenging. *Acromyrmex* ants are one such example as they almost always contain multiple strains (Van Borm et al., 2003; Andersen et al., 2012) and we do not yet have *Wolbachia* genome data. *Wsp* typing has confirmed distinct, species-specific *Wolbachia* strains for *A. echinatior* (HVR-1) and *A. insinuator* (HVR-3) as well as a shared strain between the two species and *A. octospinosus* (HVR-2; Van Borm et al., 2003; Andersen et al., 2012). Differences from this study and Van Borm et al. (2003) could mean strains are transient or that diversity is greater than what is currently known. On the other hand, differences may be related to limited ant colony sampling. Many ant species have wide geographic distributions (e.g., *Linepithema*, *Monomorium*, *Solenopsis*, *Atta*, and *Acromyrmex* genera) and show significant differences in infections among colonies and geographic locations (e.g., Reuter et al., 2005; Frost et al., 2010; Martins et al., 2012; Zhukova et al., 2017). Thus, despite previous efforts to illustrate *Wolbachia* HT events, success has been limited because we have only characterized small subpopulations and because *Wolbachia* may be evolving and spreading to new hosts faster than we currently study it.

### Part 4: Implications for Future Research

Although limited, existing data suggests *Wolbachia* associated with ants are uniquely shaped by the ant microenvironment and have occasionally taken advantage of opportunities offered by the hosts’ wide range of social interactions to “jump” to other ant species or genera. Comparisons between the widespread HVR-2 and less common strains, HVR-1 and -3, offer an exciting opportunity for future research because these strains (i) have different specificity to ant hosts (frequencies, infection levels), and (ii) have strikingly different distributions across phylogenetically distant ant hosts (although this may be driven by under-sampling). This suggests HVR-2 may have acquired (or lost) a set of genes that have facilitated its “ecological success.” Future genomic comparisons may allow us to answer important questions about *Wolbachia* evolution and HT including, why strains like HVR-2 have greater ecological success (spread), and what genes and mechanisms are associated with the ability to spread successfully across distantly related host species.

The most reliable *Wolbachia* phylogenies have been built using WGS data (Klasson et al., 2009; Ellegaard et al., 2013; Gerth et al., 2014; Gerth and Bleidorn, 2016). These phylogenies have resolved important gaps in our knowledge of *Wolbachia* origin and supergroup diversification as they are typically built using conserved orthologs unaffected by recombination, which would render topologies invalid (Gerth et al., 2014; Gerth and Bleidorn, 2016). Further mapping of *Wolbachia* diversity on host ant trees and more genomic data, particularly involving ants not hailing from the Americas, will be required to assess biogeography patterns, such as whether there are specialized *Wolbachia* lineages infecting New World ants (Russell et al., 2009; Frost et al., 2010). The existence of major consortia like the GAGA project^1^, which aims to sequence and perform comparative bacterial genomics for 200 ant genomes, shows tremendous promise for furthering knowledge of *Wolbachia* associations with a broader taxonomic host range. Comparative genomics (e.g., identification of selection signatures in genes) can shed light onto genetic prerequisites for HT. Besides advancing phylogenomic and comparative genomic approaches, WGS can provide insight into HT mechanisms for future functional studies (similar to Frydman et al., 2006; White et al., 2017) allowing us to pinpoint specific *Wolbachia* genes to relevant phenotypes.

## Author Contributions

ST performed the experiments and conducted formal analysis of the data with guidance and supervision from PS and PN. ST wrote the original draft of the manuscript. PS and PN reviewed and edited the manuscript.

## Conflict of Interest Statement

The authors declare that the research was conducted in the absence of any commercial or financial relationships that could be construed as a potential conflict of interest.

## Footnotes

1 http://antgenomics.dk/

## Supplementary Material

FIGURE S1 Relative proportion of HVR types 1–3 across *Acromyrmex* queen eggs. Pie chart showing the relative proportion of eggs sequenced from queens in this study with each HVR type, as described in Supplementary Table S1. The legend lists the HVR reference number used in this study followed by parentheses including the name of identical strains and the host species harboring those strains as described by Van Borm et al. (2003). Although closely related, the asterisk indicates that the strain Bcons was not a perfect match to HVR-2 from our study, unlike strain B1 (present in *A. insinuator* and *A. octospinosus*) which was identical to HVR-2.

TABLE S1 Top BLAST matches and corresponding HVR type for *A. echinatior* and *A. insinuator wsp* sequences generated in this study. Summary of HVR typing results from Supplementary Table S2 and additional *wsp* characterizations from the *A. echinatior* host queen (HQ) and the three parasitic *A. insinuator* (PQ) queens. From left to right: queen ant used to collect egg DNA samples for this study, host species and accession numbers for the top BLAST hits matching respective *Wolbachia* sequences, the geographic origin of NCBI samples of other ants, the average % match identity in BLAST, the number of sequences that returned these hits, HVR (hypervariable region) typing according to Baldo et al. (2006b) used in this study, and the percentage of sequences from each queen with respective HVR types. HVR typing is based on the four hypervariable regions of the *wsp* gene which are comprised of relatively conserved amino acid motifs used to identify recombination points, allowing discrimination between closely related *Wolbachia* strains, comparable to the use of antigens for serotyping pathogenic bacteria (Baldo et al., 2006b). The four HVRs occupy consecutive conserved regions of the *wsp* gene and the combination of all four HVR haplotypes make up its WSP profile. All *A. echinatior* and *A. insinuator* sequences were classified by their four HVRs using the PubMLST database ^2^ (best match to existing sequences in the database), which revealed three distinct *wsp* genotypes, here referred to as HVR 1-3. Color coding for the HVR types matches those shown in Figure 1, except for one case where sequences were chimeras (type 21-38-69-37/21-21-25-37) of HVR-1 and HVR-2. As identified in other strains, recombination was localized in the HVRs, which suggests the two *A. echinatior* sequences are true chimeras rather than sequencing errors (Andersen et al., 2012).

TABLE S2 Individual sequence information and alignment to HVR reference sequences. From left to right: colony of origin, accession numbers for sequences from this study, host species and accession numbers for the top BLAST hits matching respective *Wolbachia* sequences, % identity to BLAST match, closest match to sequences in pubMLST database (Baldo et al., 2006b) for *wsp* (nucleotide query), HVR1-4 (amino acid query) with sequence differences listed below. Colors coordinate with HVR types 1–3 used in Figure 1. Asterisks indicate sequences that differed in HVR reference number relative to other strains in the same HVR type (1–3) classified in this study. The three sequences with asterisks only exhibited a 1–3 bp difference from other sequences in their respective HVR characterization (types 1–3) and were thus included in analyses.
