# Supplementary figures and images for: Wolbachia Horizontal Transmission Events in Ants: What Do We Know and What Can We Learn?
Source: Front Microbiol. 2019 Mar 6;10:296. doi: 10.3389/fmicb.2019.00296 (PMC6414450; doi:10.3389/fmicb.2019.00296)

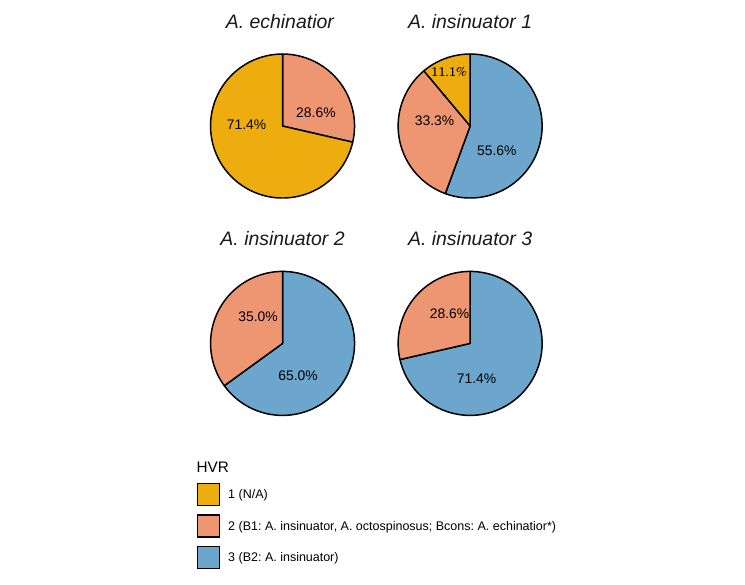

Supplement: FIGURE S1 — Relative proportion of HVR types 1–3 across Acromyrmex queen eggs. Pie chart showing the relative proportion of eggs sequenced from queens in this study with each HVR type, as described in Supplementary Table S1. The legend lists the HVR reference number used in this study followed by parentheses including the name of identical strains and the host species harboring those strains as described by Van Borm et al. (2003). Although closely related, the asterisk indicates that the strain Bcons was not a perfect match to HVR-2 from our study, unlike strain B1 (present in A. insinuator and A. octospinosus) which was identical to HVR-2. [file Image_1.TIFF]
