# Supplementary material for: Wolbachia Horizontal Transmission Events in Ants: What Do We Know and What Can We Learn?
Source: Front Microbiol. 2019 Mar 6;10:296. doi: 10.3389/fmicb.2019.00296 (PMC6414450; doi:10.3389/fmicb.2019.00296)
Supplement: TABLE S1 — Top BLAST matches and corresponding HVR type for A. echinatior and A. insinuator wsp sequences generated in this study. Summary of HVR typing results from Supplementary Table S2 and additional wsp characterizations from the A. echinatior host queen (HQ) and the three parasitic A. insinuator (PQ) queens. From left to right: queen ant used to collect egg DNA samples for this study, host species and accession numbers for the top BLAST hits matching respective Wolbachia sequences, the geographic origin of NCBI samples of other ants, the average % match identity in BLAST, the number of sequences that returned these hits, HVR (hypervariable region) typing according to Baldo et al. (2006b) used in this study, and the percentage of sequences from each queen with respective HVR types. HVR typing is based on the four hypervariable regions of the wsp gene which are comprised of relatively conserved amino acid motifs used to identify recombination points, allowing discrimination between closely related Wolbachia strains, comparable to the use of antigens for serotyping pathogenic bacteria (Baldo et al., 2006b). The four HVRs occupy consecutive conserved regions of the wsp gene and the combination of all four HVR haplotypes make up its WSP profile. All A. echinatior and A. insinuator sequences were classified by their four HVRs using the PubMLST database 2 (best match to existing sequences in the database), which revealed three distinct wsp genotypes, here referred to as HVR 1-3. Color coding for the HVR types matches those shown in Figure 1, except for one case where sequences were chimeras (type 21-38-69-37/21-21-25-37) of HVR-1 and HVR-2. As identified in other strains, recombination was localized in the HVRs, which suggests the two A. echinatior sequences are true chimeras rather than sequencing errors (Andersen et al., 2012). [file Table_1.pdf]

Queen	Most Closely Related Hosts	Accession Numbers	Geographic Origin	Average % Identity	# of Sequences	HVR Type	HVR Reference # (This Study)	% of Sequences with HVR Type
A. echinatior	*Megalomyrmex wallacei*	LC027874	Costa Rica	99.0%	15	37-38-69-37	1	71.43%
	Sericomyrmex sp. & *Neivamyrmex nigrescens*	LC027866 & KC137187	Panama, Mexico	99.5%	6	21-21-25-21	2	28.57%
	Solenopsis invicta & *Linepithema humile*	HM747152 & AY446990	Brazil	99.2%	2	21-38-69-37 / 21-21-25-37	N/A	NOT Included
A. insinuator 1	*Megalomyrmex wallacei*	LC027874	Costa Rica	98.5%	2	37-38-69-37	1	11%
	Sericomyrmex sp. & *Neivamyrmex nigrescens*	LC027866 & KC137187	Panama, Mexico	99.7%	4	21-21-25-21	2	33.33%
	Wasmannia auropunctata & *Solenopsis invicta*	JX499066 & DQ842483	French Guiana, Brazil	99.5%	2			
	Acromyrmex insinuator & *Solenopsis invicta*	AF472560 & HM747159	Panama, Brazil	99.7%	10	21-40-42-39	3	55.56%

A. insinuator 2	*Sericomyrmex sp.* & *Neivamyrmex nigrescens*	LC027866 & KC137187	Panama, Mexico	99.7%	5	21-21-25-21	2	35.0%
	Wasmannia auropunctata & *Solenopsis invicta*	JX499066 & DQ842483	French Guiana, Brazil	99.5%	2			
	Acromyrmex insinuator & *Solenopsis invicta*	AF472560 & HM747159	Panama, Brazil	99.7%	13	21-40-42-39	3	65.0%
A. insinuator 3	*Sericomyrmex sp.* & *Neivamyrmex nigrescens*	LC027866 & KC137187	Panama, Mexico	99.7%	5	21-21-25-21	2	28.57%
	Wasmannia auropunctata & *Solenopsis invicta*	JX499066 & DQ842483	French Guiana, Brazil	99.5%	1			
	Acromyrmex insinuator & *Solenopsis invicta*	AF472560 & HM747159	Panama, Brazil	99.7%	15	21-40-42-39	3	71.43%
