# Supplementary material for: Wolbachia Horizontal Transmission Events in Ants: What Do We Know and What Can We Learn?
Source: Front Microbiol. 2019 Mar 6;10:296. doi: 10.3389/fmicb.2019.00296 (PMC6414450; doi:10.3389/fmicb.2019.00296)
Supplement: TABLE S2 — Individual sequence information and alignment to HVR reference sequences. From left to right: colony of origin, accession numbers for sequences from this study, host species and accession numbers for the top BLAST hits matching respective Wolbachia sequences, % identity to BLAST match, closest match to sequences in pubMLST database (Baldo et al., 2006b) for wsp (nucleotide query), HVR1-4 (amino acid query) with sequence differences listed below. Colors coordinate with HVR types 1–3 used in Figure 1. Asterisks indicate sequences that differed in HVR reference number relative to other strains in the same HVR type (1–3) classified in this study. The three sequences with asterisks only exhibited a 1–3 bp difference from other sequences in their respective HVR characterization (types 1–3) and were thus included in analyses. [file Table_2.pdf]

Queen	Accession #	Top 3 BLAST Hits	Accession #	% Identical	pubMLST database	*wsp* typing (nucleotide query)	HVR1 (amino query)	HVR2 (amino query)	HVR3 (amino query)	HVR4 (amino query)
A. echinatior		*Megalomyrmex wallacei*	LC027874	99.4	**Closest match:**	58	37	38	69	37
		Megalomyrmex wallacei	LC027873	99.4						
	MG547498	*Megalomyrmex wallacei*	LC027872	99.4	**Differences:**	317C →T			30K →R	
		Megalomyrmex wallacei	LC027874	99.4	**Closest match:**	58	37	38	69	37
		Megalomyrmex wallacei	LC027873	99.4						
	MG547478	*Megalomyrmex wallacei*	LC027872	99.4	**Differences:**	317C →T			30K →R	
		Megalomyrmex wallacei	LC027874	99	**Closest match:**	58	37	38	69	37
		Megalomyrmex wallacei	LC027873	99		31G→A, 143G→A,				
	MG547481	*Megalomyrmex wallacei*	LC027872	99	**Differences:**	114T→G, 317C→T	11G→S	15S→K	30K →R	
		Megalomyrmex wallacei	LC027874	99.4	**Closest match:**	58	37	38	69	37
		Megalomyrmex wallacei	LC027873	99.4						
	MG547489	*Megalomyrmex wallacei*	LC027872	99.4	**Differences:**	7G→A, 317C→T	3V→I		30K→R	
		Megalomyrmex wallacei	LC027874	99.2	**Closest match:**	58	37	38	69	37
		Megalomyrmex wallacei	LC027873	99.2						
	MG547499	*Megalomyrmex wallacei*	LC027872	99.2	**Differences:**	317C→T			30K →R	
		Megalomyrmex wallacei	LC027874	99.1	**Closest match:**	58	37	38	69	37
		Megalomyrmex wallacei	LC027873	99.1						
	MG547483	*Megalomyrmex wallacei*	LC027872	99.1	**Differences:**	317C→T			30K →R	
		Megalomyrmex wallacei	LC027874	98.9	**Closest match:**	58	37	38	69	37
		Megalomyrmex wallacei	LC027873	98.9						
	MG547497	*Megalomyrmex wallacei*	LC027872	98.9	**Differences:**	317C→T,378T→C			30K→R	
		Megalomyrmex wallacei	LC027874	99	**Closest match:**	58	37	38	69	37
		Megalomyrmex wallacei	LC027873	99						
	MG547490	*Megalomyrmex wallacei*	LC027872	99	**Differences:**	91T→C,317C→T	31Y→H		30K→R	
		Megalomyrmex wallacei	LC027874	99.1	**Closest match:**	58	37	38	69	37
		Megalomyrmex wallacei	LC027873	99.1						
	MG547492	*Megalomyrmex wallacei*	LC027872	99.1	**Differences:**	317C→T			30K→R	
		Megalomyrmex wallacei	LC027874	98.8	**Closest match:**	58	30*	38	69	21*
		Megalomyrmex wallacei	LC027873	98.8		42T→G, 317C→T,				
	MG547487	*Megalomyrmex wallacei*	LC027872	98.8	**Differences:**	452T→C, 467C→A			30K→R	
		Megalomyrmex wallacei	LC027874	99.2	**Closest match:**	58	37	38	69	21*
		Megalomyrmex wallacei	LC027873	99.2						
	MG547494	*Megalomyrmex wallacei*	LC027872	99.2	**Differences:**	317C→T, 467C→A			30K→R	19A-V
		Megalomyrmex wallacei	LC027874	98.3	**Closest match:**	58	37	38	69	37
		Megalomyrmex wallacei	LC027873	98.3				2D→G,		
	MG547486	*Megalomyrmex wallacei*	LC027872	98.3	**Differences:**		29F→S	12S→L	30K→R	
		Megalomyrmex wallacei	LC027874	98.7	**Closest match:**	58	37	38	69	37
		Megalomyrmex wallacei	LC027873	98.7		85T→C,135A→C,				
	MG547480	*Megalomyrmex wallacei*	LC027872	98.7	**Differences:**	317C→T	29F →L		30K →R	
		Megalomyrmex wallacei	LC027874	98.9	**Closest match:**	58	37	38	69	37
		Megalomyrmex wallacei	LC027873	98.9						
	MG547491	*Megalomyrmex wallacei*	LC027872	98.9	**Differences:**	135A→C,317C→T			30K→R	
		Megalomyrmex wallacei	LC027874	99.2	**Closest match:**	58	37	38	69	37
		Megalomyrmex wallacei	LC027873	99.2						
	MG547495	*Megalomyrmex wallacei*	LC027872	99.2	**Differences:**	317C→T			30K→R	
		Solenopsis invicta	HM747152	98.9	**Closest match:**	58	21	38	69	37
		InvB (Linepithema humile)	AY446990	98.7		7G→A,				
						20C→A,28A→G,42T→				
	MG547493	*InvB (Linepithema humile)*	AY446989	98.7	**Differences:**	G, 105C→T, 317C→T			30K→R	
		Sericomyrmex sp.	LC027866	100	**Closest match:**	28	21	21	25	21
		Sericomyrmex sp.	LC027865	100						
	MG547500	*Neivamyrmex nigrescens*	KC137187	100	**Differences:**					
		Sericomyrmex sp.	LC027866	99.8	**Closest match:**	28	21	21	25	21
		Sericomyrmex sp.	LC027865	99.8						
	MG547482	*Neivamyrmex nigrescens*	KC137187	99.8	**Differences:**	143G→A,144T→G		15S→K		
		Sericomyrmex sp.	LC027866	99	**Closest match:**	28	21	21	25	21
		Sericomyrmex sp.	LC027865	99						
	MG547479	*Neivamyrmex nigrescens*	KC137187	99	**Differences:**			12S→L		
		Sericomyrmex sp.	LC027866	99.8	**Closest match:**	28	21	21	25	21
		Sericomyrmex sp.	LC027865	99.8						
	MG547485	*Neivamyrmex nigrescens*	KC137187	99.8	**Differences:**					
		Sericomyrmex sp.	LC027866	99.2	**Closest match:**	28	21	21	25	21
		Sericomyrmex sp.	LC027865	99.2		41A→G, 270T→C,				

MG547488	*Neivamyrmex nigrescens*	KC137187	99.2	**Differences:**	358A→G	14K→R		39K→E	
	Sericomyrmex sp.	LC027866	99.4	**Closest match:**	28	21	21	25	21
	Sericomyrmex sp.	LC027865	99.4						
MG547496	*Neivamyrmex nigrescens*	KC137187	99.4	**Differences:**	16A→G,113T→C	6I→V	5V→A		
	Solenopsis invicta	HM747152	99.8	**Closest match:**	28	21	21	25	37
	InvB (Linepithema humile)	AY446990	99.6						
MG547484	*InvB (Linepithema humile)*	AY446989	99.6	**Differences:**	452C→T,467A→C				
A. insinuator 1	*Acromyrmex insinuator 2*	AF472560	99.6	**Closest match:**	59	21	40	42	39
	Solenopsis invicta	HM747159	99.6						
MG547503	*Solenopsis invicta*	HM747156	99.6	**Differences:**	134C →T		12S→L		
	Acromyrmex insinuator 2	AF472560	99.8	**Closest match:**	59	21	40	42	39
	Solenopsis invicta	HM747159	99.8						
MG547507	*Solenopsis invicta*	HM747156	99.8	**Differences:**					
	Acromyrmex insinuator 2	AF472560	99.6	**Closest match:**	59	21	40	42	39
	Solenopsis invicta	HM747159	99.6						
MG547514	*Solenopsis invicta*	HM747156	99.6	**Differences:**	131A→T		11Y→F		
	Solenopsis invicta	HM747159	99.8	**Closest match:**	59	21	40	42	39
	Solenopsis invicta	HM747156	99.8						
MG547506	*Acromyrmex insinuator 2*	AF472560	99.6	**Differences:**					
	Solenopsis invicta	HM747159	99.8	**Closest match:**	59	21	40	42	39
	Solenopsis invicta	HM747156	99.8						
MG547508	*Acromyrmex insinuator 2*	AF472560	99.6	**Differences:**					
	Solenopsis invicta	HM747159	100	**Closest match:**	59	21	40	42	39
	Solenopsis invicta	HM747156	100						
MG547504	*Acromyrmex insinuator 2*	AF472560	100	**Differences:**					
	Solenopsis invicta	HM747159	100	**Closest match:**	59	21	40	42	39
	Solenopsis invicta	HM747156	100						
MG547518	*Acromyrmex insinuator 2*	AF472560	100	**Differences:**					
	Acromyrmex insinuator 2	AF472560	99.4	**Closest match:**	59	21	40	42	39
	Solenopsis invicta	HM747159	99.3						
MG547505	*Solenopsis invicta*	HM747156	99.3	**Differences:**	131A→T		11Y→F		
	Acromyrmex insinuator 2	AF472560	99.6	**Closest match:**	59	21	40	42	39
	Solenopsis invicta	HM747159	99.6						
MG547513	*Solenopsis invicta*	HM747156	99.6	**Differences:**	127C→G		10L→V		
	Acromyrmex insinuator 2	AF472560	99.6	**Closest match:**	59	21	40	42	39
	Solenopsis invicta	HM747159	99.6						
MG547515	*Solenopsis invicta*	HM747156	99.6	**Differences:**	413T→C				8F→S
	Sericomyrmex sp.	LC027866	99.6	**Closest match:**	28	21	21	25	21
	Sericomyrmex sp.	LC027865	99.6						
MG547517	*Neivamyrmex nigrescens*	KC137187	99.6	**Differences:**	261A→G				
	Sericomyrmex sp.	LC027866	100	**Closest match:**	28	21	21	25	21
	Sericomyrmex sp.	LC027865	100						
MG547511	*Neivamyrmex nigrescens*	KC137187	100	**Differences:**					
	Sericomyrmex sp.	LC027866	99.8	**Closest match:**	28	21	21	25	21
	Sericomyrmex sp.	LC027865	99.8						
MG547501	*Neivamyrmex nigrescens*	KC137187	99.8	**Differences:**					
	Wasmannia auropunctata	JX499066	99.8	**Closest match:**	28	21	21	25	21
	Solenopsis invicta	DQ842483	99.8						
MG547509	*Solenopsis invicta*	HM747152	99.8	**Differences:**	261A→G				
	Wasmannia auropunctata	JX499066	99.2	**Closest match:**	28	21	21	25	21
	Solenopsis invicta	DQ842483	99.2						
MG547512	*Solenopsis invicta*	HM747152	99.1	**Differences:**	133T→C, 135A→C, 366T→C		12S→P		
	Sericomyrmex sp.	LC027866	99.2	**Closest match:**	28	21	21	25	21
	Sericomyrmex sp.	LC027865	99.2						
MG547502	*Neivamyrmex nigrescens*	KC137187	99.2	**Differences:**	135A→C, 137A→C, 322A→G		13Q→P	27K→E	
	Megalomyrmex wallacei	LC027874	98.5	**Closest match:**	58	37	38	69	37
	Megalomyrmex wallacei	LC027873	98.5						
MG547510	*Megalomyrmex wallacei*	LC027872	98.5	**Differences:**	28A→G, 90T→C, 166A→G, 204T→C, 317C→T	10T→A	23T→A	30K→R	
	Megalomyrmex wallacei	LC027874	98.5	**Closest match:**	58	37	38	69	37
	Megalomyrmex wallacei	LC027873	98.5						
MG547516	*Megalomyrmex wallacei*	LC027872	98.5	**Differences:**	28A→G, 135A→C, 166A→G, 204T→C, 317C→T	10T→A	23T→A	30K →R	
A. insinuator 2	*Acromyrmex insinuator 2*	AF472560	99.8	**Closest match:**	59	21	40	42	39
	Solenopsis invicta	HM747159	99.8						
MG547529	*Solenopsis invicta*	HM747156	99.8	**Differences:**					

	Acromyrmex insinuator 2	AF472560	99.6	**Closest match:**	59	21	40	42	39
	Solenopsis invicta	HM747159	99.6						
MG547531	*Solenopsis invicta*	HM747156	99.6	**Differences:**	214T→C		39Y→H		
	Acromyrmex insinuator 2	AF472560	99.8	**Closest match:**	59	21	40	42	39
	Solenopsis invicta	HM747159	99.8						
MG547535	*Solenopsis invicta*	HM747156	99.8	**Differences:**	107T→A		3I→N		
	Solenopsis invicta	HM747159	100	**Closest match:**	59	21	40	42	39
	Solenopsis invicta	HM747156	100						
MG547538	*Acromyrmex insinuator 2*	AF472560	100	**Differences:**					
	Acromyrmex insinuator 2	AF472560	99.8	**Closest match:**	59	21	40	42	39
	Solenopsis invicta	HM747159	99.8						
MG547519	*Solenopsis invicta*	HM747156	99.8	**Differences:**					
	Acromyrmex insinuator 2	AF472560	99.6	**Closest match:**	59	21	40	42	39
	Solenopsis invicta	HM747159	99.6						
MG547530	*Solenopsis invicta*	HM747156	99.6	**Differences:**	127C→G		10L→V		
	Solenopsis invicta	HM747159	99.3	**Closest match:**	59	21	40	42	39
	Solenopsis invicta	HM747156	99.3						
MG547533	*Acromyrmex insinuator 2*	AF472560	99.2	**Differences:**	78T→C, 127C→G		10L→V		
	Solenopsis invicta	HM747159	100	**Closest match:**	59	21	40	42	39
	Solenopsis invicta	HM747156	100						
MG547537	*Acromyrmex insinuator 2*	AF472560	100	**Differences:**					
	Solenopsis invicta	HM747159	99.8	**Closest match:**	59	21	40	42	39
	Solenopsis invicta	HM747156	99.8						
MG547524	*Acromyrmex insinuator 2*	AF472560	99.8	**Differences:**					
	Solenopsis invicta	HM747159	99.6	**Closest match:**	59	21	40	42	39
	Solenopsis invicta	HM747156	99.6						
MG547532	*Solenopsis invicta*	JQ425789	99.3	**Differences:**	348A→G, 444A→G				
	Solenopsis invicta	HM747159	99.8	**Closest match:**	59	21	40	42	39
	Solenopsis invicta	HM747156	99.8						
MG547526	*Acromyrmex insinuator 2*	AF472560	99.6	**Differences:**					
	Solenopsis invicta	HM747159	99.6	**Closest match:**	59	21	40	42	39
	Solenopsis invicta	HM747156	99.6						
MG547527	*Acromyrmex insinuator 2*	AF472560	99.4	**Differences:**	69A→G	23I→M			
	Acromyrmex insinuator 2	AF472560	99.2	**Closest match:**	59	21	40	42	39
	Solenopsis invicta	HM747159	99.1		127C→G, 135A→G,				
MG547523	*Solenopsis invicta*	HM747156	99.1	**Differences:**	320A→G		10L→V	29K→R	
	Sericomyrmex sp.	LC027866	99.8	**Closest match:**	28	21	21	25	21
	Sericomyrmex sp.	LC027865	99.8						
MG547520	*Neivamyrmex nigrescens*	KC137187	99.8	**Differences:**					
	Sericomyrmex sp.	LC027866	99.6	**Closest match:**	28	21	21	25	21
	Sericomyrmex sp.	LC027865	99.6						
MG547534	*Neivamyrmex nigrescens*	KC137187	99.6	**Differences:**	135A→C				
	Sericomyrmex sp.	LC027866	99.8	**Closest match:**	28	21	21	25	21
	Sericomyrmex sp.	LC027865	99.8						
MG547521	*Neivamyrmex nigrescens*	KC137187	99.8	**Differences:**					
	Sericomyrmex sp.	LC027866	99.6	**Closest match:**	28	21	21	25	21
	Sericomyrmex sp.	LC027865	99.6						
MG547536	*Neivamyrmex nigrescens*	KC137187	99.6	**Differences:**	69A→G	23I→M			
	Sericomyrmex sp.	LC027866	99.6	**Closest match:**	28	21	21	25	21
	Sericomyrmex sp.	LC027865	99.6						
MG547525	*Neivamyrmex nigrescens*	KC137187	99.6	**Differences:**	60A→G				
	Wasmannia auropunctata	JX499066	99.4	**Closest match:**	28	21	21	25	21
	Solenopsis invicta	DQ842483	99.4						
MG547528	*Solenopsis invicta*	HM747152	99.3	**Differences:**	135A→C, 137A→C		13Q→P		
	Wasmannia auropunctata	JX499066	99.6	**Closest match:**	28	21	21	25	21
	Solenopsis invicta	DQ842483	99.6						
MG547522	*Solenopsis invicta*	HM747152	99.6	**Differences:**	243A→G				
A. insinuator 3	*Acromyrmex insinuator 2*	AF472560	99.6	**Closest match:**	59	21	40	42	39
	Solenopsis invicta	HM747159	99.6						
MG547539	*Solenopsis invicta*	HM747156	99.6	**Differences:**					
	Solenopsis invicta	HM747159	99.8	**Closest match:**	59	21	40	42	39
	Solenopsis invicta	HM747156	99.8						
MG547552	*Acromyrmex insinuator 2*	AF472560	99.6	**Differences:**					
	Solenopsis invicta	HM747159	99.8	**Closest match:**	59	21	40	42	39
	Solenopsis invicta	HM747156	99.8						
MG547550	*Acromyrmex insinuator 2*	AF472560	99.6	**Differences:**					
	Acromyrmex insinuator 2	AF472560	99.6	**Closest match:**	59	21	40	42	39

	Solenopsis invicta	HM747159	99.6						
MG547555	*Solenopsis invicta*	HM747156	99.6	**Differences:**	401G→A				4G→D
	Acromyrmex insinuator 2	AF472560	99.8	**Closest match:**	59	21	40	42	39
	Solenopsis invicta	HM747159	99.8						
MG547541	*Solenopsis invicta*	HM747156	99.8	**Differences:**					
	Acromyrmex insinuator 2	AF472560	99.8	**Closest match:**	59	21	40	42	39
	Solenopsis invicta	HM747159	99.8						
MG547556	*Solenopsis invicta*	HM747156	99.8	**Differences:**					
	Acromyrmex insinuator 2	AF472560	99.6	**Closest match:**	59	21	40	42	39
	Solenopsis invicta	HM747159	99.6						
MG547548	*Solenopsis invicta*	HM747156	99.6	**Differences:**	243T→C				
	Solenopsis invicta	HM747159	100	**Closest match:**	59	21	40	42	39
	Solenopsis invicta	HM747156	100						
MG547559	*Acromyrmex insinuator 2*	AF472560	100	**Differences:**					
	Solenopsis invicta	HM747159	100	**Closest match:**	59	21	40	42	39
	Solenopsis invicta	HM747156	100						
MG547549	*Acromyrmex insinuator 2*	AF472560	100	**Differences:**					
	Solenopsis invicta	HM747159	99.8	**Closest match:**	59	21	40	42	39
	Solenopsis invicta	HM747156	99.8						
MG547546	*Acromyrmex insinuator 2*	AF472560	99.6	**Differences:**					
	Solenopsis invicta	HM747159	99.3	**Closest match:**	59	21	40	42	39
	Solenopsis invicta	HM747156	99.3						
MG547547	*Acromyrmex insinuator 2*	AF472560	99.2	**Differences:**	36A→G, 270T→C				
	Acromyrmex insinuator 2	AF472560	99.6	**Closest match:**	59	21	40	42	39
	Solenopsis invicta	HM747159	99.6						
MG547545	*Solenopsis invicta*	HM747156	99.6	**Differences:**	186A→G				
	Acromyrmex insinuator 2	AF472560	99.6	**Closest match:**	59	21	40	42	39
	Solenopsis invicta	HM747159	99.6						
MG547540	*Solenopsis invicta*	HM747156	99.6	**Differences:**	30A→T				
	Acromyrmex insinuator 2	AF472560	99.4	**Closest match:**	59	21	40	42	39
	Solenopsis invicta	HM747159	99.3						
MG547551	*Solenopsis invicta*	HM747156	99.3	**Differences:**					
	Acromyrmex insinuator 2	AF472560	99.2	**Closest match:**	59	21	40	42	39
	Solenopsis invicta	HM747159	99.1		15T→C, 127C→G,				
MG547553	*Solenopsis invicta*	HM747156	99.1	**Differences:**	241C→T		10L→V	3P→S	
	Sericomyrmex sp.	LC027866	99.6	**Closest match:**	28	21	21	25	21
	Sericomyrmex sp.	LC027865	99.6						
MG547543	*Neivamyrmex nigrescens*	KC137187	99.6	**Differences:**	135A→C				
	Sericomyrmex sp.	LC027866	99.8	**Closest match:**	28	21	21	25	21
	Sericomyrmex sp.	LC027865	99.8						
MG547554	*Neivamyrmex nigrescens*	KC137187	99.8	**Differences:**					
	Sericomyrmex sp.	LC027866	100	**Closest match:**	28	21	21	25	21
	Sericomyrmex sp.	LC027865	100						
MG547557	*Neivamyrmex nigrescens*	KC137187	100	**Differences:**					
	Sericomyrmex sp.	LC027866	99.8	**Closest match:**	28	21	21	25	21
	Sericomyrmex sp.	LC027865	99.8						
MG547542	*Neivamyrmex nigrescens*	KC137187	99.8	**Differences:**					
	Wasmannia auropunctata	JX499066	99.6	**Closest match:**	28	21	21	25	21
	Solenopsis invicta	DQ842483	99.6						
MG547544	*Solenopsis invicta*	HM747152	99.6	**Differences:**	441T→441C				
	Sericomyrmex sp.	LC027866	99.6	**Closest match:**	28	21	21	261*	21
	Sericomyrmex sp.	LC027865	99.6					29Q-L,	
MG547558	*Neivamyrmex nigrescens*	KC137187	99.6	**Differences:**	329A-T, 377A-G			30K-R	
